# SARS‐CoV‐2 infection and new‐onset type 1 diabetes in the post‐acute period among children and young people in England

**DOI:** 10.1111/dme.70084

**Published:** 2025-06-17

**Authors:** Joseph L. Ward, Joana Cruz, Rachel Harwood, Simon Kenny, Dougal Hargreaves, Kamlesh Khunti, Jonathan Valabhji, Bianca De Stavola, Russell M. Viner

**Affiliations:** ^1^ Department of Women and Children's Health King's College London London UK; ^2^ UCL Great Ormond St. Institute of Child Health London UK; ^3^ Institute of Systems, Molecular and Integrative Biology, University of Liverpool Liverpool UK; ^4^ NHS England London UK; ^5^ Imperial College London, Mohn Centre for Children's Health & Wellbeing London UK; ^6^ Diabetes Research Centre, University of Leicester, Leicester General Hospital Leicester UK; ^7^ Department of Metabolism, Digestion and Reproduction, Faculty of Medicine Chelsea and Westminster Hospital Campus, Imperial College London London UK

**Keywords:** children and young people, epidemiology, population cohort, SARS‐CoV‐2, type 1 diabetes

## Abstract

**Aims:**

To examine if SARS‐CoV‐2 infection is associated with new‐onset type 1 diabetes in the post‐acute period in children and young people (CYP).

**Methods:**

In this population cohort, we used data on all hospital activity in England to estimate type 1 diabetes incidence among CYP aged 0–17 exposed to SARS‐CoV‐2 between May 2020 and August 2022, from day 28 after a positive test for the following 6 months. We compared this with unexposed CYP who were hospitalized for elective procedures or following trauma during the pandemic, and in the 2 years prior to the pandemic (historic cohorts). We excluded CYP with prior chronic illnesses. We undertook Cox regression analyses adjusted for age, sex, ethnicity, deprivation and season of index date, and stratified by periods when different SARS‐CoV‐2 variants were dominant.

**Results:**

There were 1,087,604 CYP in the exposed cohort, 143,748 in the trauma cohort, 253,368 in the elective cohort, 160,925 in the historic trauma cohort and 388,673 in the historic elective cohort. Hazard of developing type 1 diabetes was significantly higher among those exposed than unexposed CYP: 2.4 [1.58–3.64] relative to the trauma cohort, 2.9 [2.00–4.13] relative to the elective cohort, 4.2 [2.56–7.04] relative to the historic trauma cohort and 2.4 [1.81–3.10] relative to the historic elective cohort. Associations may be strongest during the Delta period.

**Conclusions:**

SARS‐CoV‐2 infection is associated with subsequent incident type 1 diabetes in the 1–7 months after an acute infection in previously healthy CYP.

**What's new?**

- The incidence of type 1 diabetes increased in children and young people during the COVID‐19 pandemic, but the role of SARS‐CoV‐2 infection in this remains unclear.
- We undertook a population cohort study using multiple linked datasets in England.
- In adjusted cox regression models, the hazard for developing new‐onset type 1 diabetes was significantly higher in the 6 months after a SARS‐CoV‐2 infection than in CYP who did not have a positive SARS‐CoV‐2 test.
- SARS‐CoV‐2 infection is associated with new‐onset type 1 diabetes.
- This may impact future burden and should prompt further study to understand the mechanisms behind this association.

There is evidence that the incidence of type 1 diabetes increased in children and young people (CYP) during the COVID‐19 pandemic,^1^, ^2^, ^3^, ^4^ and that presentations with diabetic ketoacidosis were more common.^2^, ^5^, ^6^ The RCPCH National Paediatric Diabetes Audit, which collects data from all Paediatric Diabetes Units in England and Wales, reported a 20%–25% increase in the incidence rate for type 1 diabetes in 2020/2021 compared with 2019/2020, on a background of stable incidence rates for several years prior to this.^7^ As the pandemic subsided, the incidence of type 1 diabetes in CYP also declined.^8^, ^9^ However, how these trends are associated with SARS‐CoV‐2 exposure is unclear.

A range of viral infections are thought to be associated with developing diabetes or precipitating onset.^10^, ^11^, ^12^, ^13^, ^14^ Although there is clinical and laboratory evidence to suggest SARS‐CoV‐2 infection may itself trigger diabetes, or accelerate onset in CYP who are predisposed,^15^ the mechanisms for this are not fully understood.^14^, ^16^, ^17^ Some epidemiological studies have reported increases in new‐onset diabetes after SARS‐CoV‐2 infection both in the acute and post‐acute period in adults.^18^, ^19^, ^20^, ^21^, ^22^, ^23^, ^24^ Studies examining this association in CYP have been mixed, with data from the US Centers for Disease Control and Prevention (CDC)^25^ and others^22^, ^26^, ^27^, ^28^ finding an increase in type 1 diabetes incidence 30 days after SARS‐CoV‐2 infection, but other work finding no association.^29^, ^30^, ^31^, ^32^ Establishing if SARS‐CoV‐2 infection is associated with an increased risk of developing type 1 diabetes is important as this may have consequences for future disease burden and prevention strategies in CYP.

Previous work in this area has been limited by the lack of asymptomatic controls, use of regional or hospital‐based data, small sample sizes, and uncertainty around date and type of diabetes diagnosis. Here we use a national dataset containing all hospital activity in England from 2015, linked to SARS‐CoV‐2 testing data and date and type of all diabetes diagnoses in CYP. In this population cohort study, we use these unique linked datasets to investigate the association between post‐acute SARS‐CoV‐2 infection and new‐onset type 1 diabetes in CYP in England during the first 2 years of the pandemic. Similar to previous work,^22^, ^25^, ^26^, ^29^ we chose to investigate these associations in the post‐acute period. Analysing type 1 diabetes incidence during an acute infection is complicated by increased SARS‐CoV‐2 testing as symptoms of diabetes emerge.^29^ Further, as the mean duration of symptoms prior to diabetes diagnosis is over 3 weeks,^33^ many CYP diagnosed during an acute illness may have had diabetes prior to infection.^29^

## 1 RESEARCH DESIGN AND METHODS

### 1.1 Data

We used Secondary Use Services (SUS) data, containing sociodemographic characteristics and clinical details of all individuals admitted to hospital in England from March 2015 to August 2022. SUS data were deterministically linked to the following: (1) the National Diabetes Audit^34^ (NDA), providing type and date of diabetes diagnosis; (2) SARS‐CoV‐2 positive test data held by NHS England (date of positive polymerase chain reaction and lateral flow tests in the community and in hospital); (3) mortality data from the Office for National Statistics and National Child Mortality Database.

### 1.2 Population

CYP aged 0–17 were eligible for inclusion in this study if they had been admitted to hospital in England for any reason at any time from 1 March 2015 to 31 August 2022 (i.e. were present in the SUS data, and so could be linked to the other datasets). Note it is standard practice in England to hospitalise all CYP newly diagnosed with type 1 diabetes in order to support establishing treatment.

### 1.3 Exposed Cohort

We defined the exposed cohort as all CYP with a first positive SARS‐CoV‐2 test between 1 May 2020 and 31 August 2022 who were also present within the SUS data. We defined the cohort inception date (index date) as the first positive test date during this time. We categorized these infections according to the predominant SARS‐CoV‐2 variants in the United Kingdom as Wild type (1 May–7 December 2020), Alpha (8 December 2020–17 May 2021), Delta (18 May 2021–13 December 2021) and Omicron (BA.1, BA.2, BA.4, BA.5) (14 December 2021–31 August 2022).^35^

### 1.4 Unexposed cohorts

We identified four unexposed cohorts in our analysis. We did not have access to negative SARS‐CoV‐2 test data. However, as all admissions to hospital in England were routinely tested for SARS‐CoV‐2 immediately before admission from the end of April 2020 to 31 August 2022,^36^ we used admission dates within CYP who had not tested positive for SARS‐CoV‐2 during this period as a proxy for negative tests (pandemic cohorts). We only included elective and traumatic admissions in these cohorts to capture otherwise healthy CYP in our analysis. We also examined type 1 diabetes incidence among CYP following elective and traumatic admissions in the 2 years prior to the pandemic (historic cohorts) for comparison. CYP included in the pandemic cohorts were excluded from the historic cohorts. The unexposed cohorts were defined as follows:

1. Trauma unexposed cohorts
  - Pandemic—CYP admitted to hospital with a primary diagnosis of trauma between 1 May 2020 and 31 August 2022 and not had any positive SARS‐CoV‐2 test during this time.
  - Historic—Admitted to hospital with a primary diagnosis of trauma between 1 January 2018 and 31 December 2019.

We defined the index date as the first traumatic admission date during these time periods.

2 Elective unexposed cohorts
  - Pandemic—CYP admitted to hospital electively between 1 May 2020 and 31 August 2022 and not had any positive SARS‐CoV‐2 tests during this time.
  - Historic—Admitted to hospital electively between 1 January 2018 and 31 December 2019.

We defined the index date as the first elective admission date during these time periods.

### 1.5 Exclusions

We excluded all those with any chronic medical problems recorded within SUS prior to each cohort index date, using established code lists (see Table S1). We did this in order to compare the incidence of type 1 diabetes in otherwise healthy CYP, without conditions that may be associated with its onset.

### 1.6 Outcomes

We identified CYP who developed type 1 diabetes using the date of diagnosis and type of diabetes recorded within the NDA (type 1, type 2 or unknown). We report the overall incidence of new‐onset type 1 diabetes within each cohort and at three time points from the index date: 0–27 days (acute period), 28–209 days (i.e. 6 months after Day 28—post‐acute period) and 210 days or longer (late period). However, our primary outcome of interest was new‐onset type 1 diabetes in the post‐acute period (28–209 days).

### 1.7 Follow‐up

We defined the index date as either date of first positive SARS‐CoV‐2 test (exposed cohort), date of first traumatic admission within each period (traumatic cohorts) or date of first elective admission within each period (elective cohorts). Follow‐up time for the survival analysis was defined as starting from 28 days after index date to the first of either: 210 days (6 months after day 28), date of death, 31 August 2022 (pandemic cohorts), 31 December 2019 (historic cohorts) or date of type 1 diabetes diagnosis. We also censored CYP who developed T2DM and diabetes where type was undetermined during follow‐up.

### 1.8 Covariates

Covariates were limited to data available in the SUS dataset. We used hospitalisation data from 1 March 2015 onwards to identify previously medically recorded co‐morbidities in CYP and to populate sociodemographic variables. We categorised age as follows: infants, 1–9 and 10–17 years, and ethnicity as follows: White, Mixed, Asian, Black, Other and unknown. We used population weighted Index of Multiple Deprivation (IMD) 2019 quintile category (hereafter IMD category) to define area level socioeconomic status using address at most recent hospitalisation. We included season of index date as a covariate to account for variation throughout the year in new‐onset type 1 diabetes, SARS‐CoV‐2 infections, traumatic injuries and elective admissions (see Figures S1–S4). We coded this as follows: Spring (March, April, May), Summer (June, July, August), Autumn (September, October, November) and Winter (December, January, February).

### 1.9 Analysis

We first describe the incidence rate of type 1 diabetes during follow‐up among CYP exposed to SARS‐CoV‐2 and those not exposed from Day 0 to Day 27 (acute period), Day 28–Day 209 (post‐acute period) and from day 210 onwards (late period). We produced survival curves to show the probability of developing type 1 diabetes during the post‐acute follow‐up period by exposure and cohort. We then used Cox regression survival analyses to compare the relative hazard of developing type 1 diabetes within CYP exposed and unexposed to SARS‐CoV‐2, adjusted for age group, sex, ethnicity and IMD quintile and index date season. We examined the association between the exposed cohort and each of the unexposed cohorts separately (note CYP could be in both the elective and traumatic unexposed cohorts). We then examined these associations according to the dominant SARS‐CoV‐2 variant in England at the time CYP entered each cohort. We undertook the analysis in STATA 16, using the command *stcox*. We assessed the assumption of proportional hazards by examining trends in Schoenfeld's residuals using the command *stphtest*.

## 2 RESULTS

We identified 1,087,604 CYP in the exposed cohort, 143,748 in the trauma cohort, 253,368 in the elective cohort, 160,925 in the historic trauma cohort and 388,673 in the historic elective cohort (Table 1). There were higher proportions of CYP who were female, White, aged 1–9 and in the least deprived quintile in the exposed cohort compared with the unexposed cohorts. The most common reasons for admission within the elective cohorts were for dental, ear, nose and throat and ophthalmological conditions; within the traumatic cohorts, hospitalisations were predominantly for limb fractures, open wounds (lacerations) and poisonings. Within CYP exposed to SARS‐CoV‐2, the most common reasons for being within the SUS dataset (and so available for inclusion in the study) were admissions related to birth and the newborn period, acute viral infections, and dental caries (see Tables S2–S6). Across all cohorts, 2637 CYP were diagnosed with type 1 diabetes during follow‐up, 395 with type 2 diabetes and 324 with diabetes where type was unknown.

**TABLE 1:** Demographic characteristics of CYP in each cohort.

	Pandemic 1 May 2020–31 August 2022						Pre‐pandemic 1 January 2018–January 2020			
	Exposed		Traumatic^a^		Elective^b^		Traumatic^c^		Elective^d^	
	*n*	(%)	*n*	(%)	*n*	(%)	*n*	(%)	*n*	(%)
Total	1,087,604		143,748		253,368		160,925		388,673	
Sex										
Female	528,938	(48.6)	62,450	(43.4)	109,348	(43.2)	68,941	(42.8)	172,784	(44.5)
Male	558,666	(51.4)	81,298	(56.6)	144,020	(56.8)	91,984	(57.2)	215,889	(55.5)
Age										
<1	46,257	(4.3)	14,130	(9.8)	32,382	(12.8)	13,153	(8.2)	31,499	(8.1)
1–9	663,434	(61.0)	74,036	(51.5)	136,234	(53.8)	75,313	(46.8)	203,024	(52.2)
10–17	377,913	(34.7)	55,582	(38.7)	84,752	(33.5)	72,459	(45.0)	154,150	(39.7)
Ethnicity										
White	815,728	(75.0)	100,872	(70.2)	158,252	(62.5)	114,736	(71.3)	252,527	(65.0)
Black	22,335	(2.1)	6096	(4.2)	12,337	(4.9)	6499	(4.0)	16,237	(4.2)
Asian	77,524	(7.1)	12,507	(8.7)	25,017	(9.9)	13,633	(8.5)	35,498	(9.1)
Mixed	43,967	(4.0)	6290	(4.4)	11,178	(4.4)	6050	(3.8)	14,098	(3.6)
Other	26,354	(2.4)	5011	(3.5)	9386	(3.7)	5444	(3.4)	12,708	(3.3)
Unknown	101,696	(9.4)	12,972	(9.0)	37,198	(14.7)	14,563	(9.0)	57,605	(14.8)
IMD^e^ quintile										
Most deprived	187,896	(17.3)	34,990	(24.3)	58,137	(22.9)	39,279	(24.4)	88,126	(22.7)
Second most deprived	199,852	(18.4)	29,881	(20.8)	54,569	(21.5)	33,377	(20.7)	83,136	(21.4)
Third most deprived	221,547	(20.4)	28,025	(19.5)	49,719	(19.6)	31,489	(19.6)	76,804	(19.8)
Fourth most deprived	234,900	(21.6)	26,566	(18.5)	47,389	(18.7)	29,687	(18.4)	72,478	(18.6)
Least deprived	241,507	(22.2)	24,283	(16.9)	43,550	(17.2)	27,090	(16.8)	68,123	(17.5)
Missing	1902	(0.2)	3	(0.0)	4	(0.0)	3	(0.0)	6	(0.0)

^a^ CYP admitted due to trauma 1 May 2020–31 August 2022 and not testing positive for SARS‐CoV‐2.

^b^ CYP admitted electively 1 May 2020–31 August 2022 and not testing positive for SARS‐CoV‐2.

^c^ CYP admitted due to trauma 1 January 2018–1 January 2020.

^d^ CYP admitted electively 1 January 2018–1 January 2020.

^e^ Index of multiple deprivation.

**FIGURE 1:**
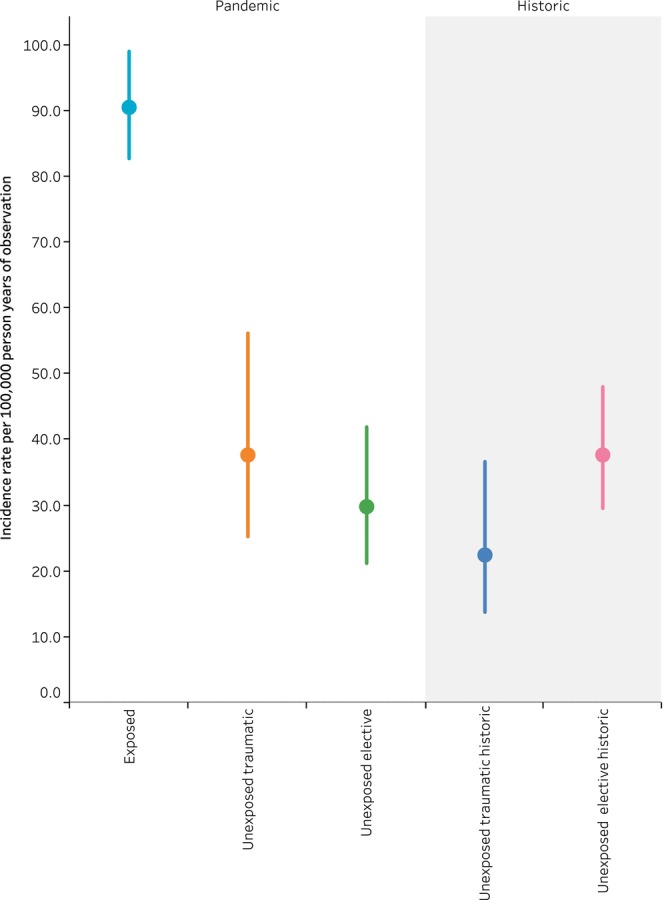
Incidence rate of new‐onset type 1 diabetes from Day 28 to Day 208 of follow‐up per 100,000 person‐years of observation. Incident rate ratios per 100,000 for being diagnosed with T1DM from Day 28 to Day 208 amongst each cohort. Exposed: CYP testing positive for SARS‐CoV‐2; unexposed traumatic: CYP admitted due to trauma 1 May 2020–31 August 2022 and not testing positive for SARS‐CoV‐2; unexposed elective: CYP admitted electively 1 May 2020–31 August 2022 and not testing positive for SARS‐CoV‐2; unexposed elective traumatic: CYP admitted due to trauma 1 January 2018–1 January 2020; unexposed elective historic: CYP admitted electively 1 January 2018–1 January 2020.

Table 2 and Figure 1 show the number and incidence rate per 100,000 person years of observation for developing type 1 diabetes during follow‐up within each cohort. Within the exposed cohort, 475 CYP developed type 1 diabetes from Day 28 to Day 209, IR 90.5 [82.7–99.0]. This compared with 24 in the trauma cohort (IR 37.6 [25.2–56.2]), 33 in the elective cohort (IR 29.8 [21.2–41.9]), 16 in the historic trauma cohort (IR 22.4 [13.8–36.6]) and 65 in the historic elective cohort (IR 37.7 [29.5–48.0]). Graphs showing the probability of developing new‐onset type 1 diabetes from Day 28 to Day 209 are shown in Figure S5.

**TABLE 2:** Incidence rate of new‐onset type 1 diabetes per 100,000 person‐years of observation within each cohort by follow‐up time.

	At any time			Less than 28 days			28 days to 208 days			Longer than 208 days		
	n	IR^e^	95% CI	n	IR^e^	95% CI	n	IR^e^	95% CI	n	IR^e^	95% CI
Pandemic period												
Exposed to SARS‐CoV‐2	944	102.9	[96.6–109.7]	220	254.8	[223.3–290.8]	475	90.5	[82.7–99.0]	249	79.6	[70.3–90.1]
Unexposed traumatic^a^ admissions	58	34.5	[26.6–44.6]	3	26.7	[8.6–82.8]	24	37.6	[25.2–56.2]	31	32.1	[22.6–45.7]
Unexposed elective^b^ admissions	115	43.1	[35.9–51.7]	46	232.9	[174.5–310.9]	33	29.8	[21.2–41.9]	36	25.2	[18.2–35.0]
Pre‐pandemic period												
Unexposed traumatic^c^ historic	38	23.0	[16.7–31.6]	3	23.7	[7.7–73.6]	16	22.4	[13.8–36.6]	19	22.4	[14.3–35.1]
Unexposed elective^d^ historic	184	45.1	[39.0–52.1]	40	131.0	[96.1–178.6]	65	37.7	[29.5–48.0]	79	36.9	[29.6–46.0]

^a^ CYP admitted due to trauma 1 May 2020–31 August 2022 and not testing positive for SARS‐CoV‐2.

^b^ CYP admitted electively 1 May 2020–31 August 2022 and not testing positive for SARS‐CoV‐2.

^c^ CYP admitted due to trauma 1 January 2018–1 January 2020.

^d^ CYP admitted electively 1 January 2018–1 January 2020.

^e^ Incident rate per 100,000 person‐years.

Table 3 shows adjusted hazard ratios from cox regression models for being diagnosed with type 1 diabetes from day 28 to Day 209 of follow‐up within the exposed and unexposed cohorts. After adjusting for age, sex, ethnicity, IMD quintile and index date season, hazard ratios for developing type 1 diabetes in CYP exposed to SARS‐CoV‐2 were significantly higher compared with each unexposed cohort, and were: 2.4 [1.58–3.64] *p* < 0.001 compared with the trauma cohort, 2.9 [2.00–4.13] compared with the elective cohort, 4.2 [2.56–7.04] compared with the historic trauma cohort and 2.4 [1.81–3.10] for the historic elective cohort. We did not find evidence against the proportional hazard assumption in any of the fitted models (*p* > 0.05).

**TABLE 3:** Hazard ratio of developing new‐onset diabetes from Day 28 to Day 208 in the exposed cohort compared with each unexposed cohort.

	Hazard ratio	95% CI		*p*	ph Test
Pandemic period					
Unexposed traumatic^a^	1				
Exposed^e^	2.4	1.58	3.64	<0.001	0.42
Unexposed elective^b^	1				
Exposed	2.9	2.00	4.13	<0.001	0.40
Pre‐pandemic period					
Unexposed traumatic^c^ (historic)	1				
Exposed	4.2	2.56	7.04	<0.001	0.28
Unexposed elective^d^ (historic)	1				
Exposed	2.4	1.81	3.10	<0.001	0.38

^a^ CYP admitted due to trauma 1 May 2020–31 August 2022 and not testing positive for SARS‐CoV‐2.

^b^ CYP admitted electively 1 May 2020–31 August 2022 and not testing positive for SARS‐CoV‐2.

^c^ CYP admitted due to trauma 1 January 2018–1 January 2020.

^d^ CYP admitted electively 1 January 2018–1 January 2020.

^e^ CYP exposed to SARS‐CoV‐2. Models adjusted for sex, age, ethnicity, Index of multiple deprivation quintile, index date season.

Tables S8 and S9 and Figure S6 show the number of CYP and incidence rate for developing type 1 diabetes according to which SARS‐CV‐2 variant was dominant at the time CYP entered the study. Analyses by variant were limited due to small numbers and wide confidence intervals. In the exposed cohort, the incidence rate per 100,000 years of observation for being diagnosed with type 1 diabetes from day 28 to day 209 was highest during the delta period (IR 144.6 [101.4–129.6]), although confidence intervals overlapped those during the Wild Type period (Figure S6). In contrast, incidence rates for developing type 1 diabetes within the unexposed elective and traumatic cohorts were similar among CYP who entered the study when each SARS‐CoV‐2 variant was dominant.

After adjusting for sex, age, ethnicity, IMD quintile and index date season, the hazard ratio for being diagnosed with type 1 diabetes from day 28 to day 209 was only significantly higher in CYP exposed to SARS‐CoV‐2 compared with CYP in the unexposed traumatic cohort during the Delta period (HR 2.6 [1.24–5.65], *p* = 0.01). Adjusted hazard ratios for being diagnosed with type 1 diabetes among CYP exposed to SARS‐CoV‐2 compared with CYP in the unexposed elective cohort were significantly higher during Wild Type (HR 1.26 [1.26–8.01]. *p* = 0.01), Delta (HR 3.5 [1.82–6.56], *p* < 0.001) and Omicron (HR 4.3 [1.52–11.88], *p* = 0.01) periods, (Table S10). We did not find evidence against the proportional hazard assumption in any of the fitted models (*p* > 0.05).

## 3 CONCLUSIONS

In this population cohort study of previously well CYP in England, we found exposure to SARS‐CoV‐2 was associated with a significantly higher incidence of developing type 1 diabetes compared with CYP who were not exposed. Although the absolute numbers of CYP being diagnosed with type 1 diabetes remained low in all cohorts, relative increases were large. We found a 2.4‐fold increase in diabetes incidence in the first 6‐month period after an acute infection with SARS‐CoV‐2, after adjusting for sociodemographic variables and seasonality. There was some evidence this association was weakest during the Alpha period and strongest during the Delta, but analyses by dominant variant were limited by small numbers and wide confidence intervals.

### 3.1 Comparison with previous literature

Our findings support previous analyses demonstrating increased incidence of type 1 diabetes after SARS‐CoV‐2 infection in both adults^18^, ^19^, ^20^, ^21^, ^22^ and CYP.^22^, ^25^, ^26^ Barrett et al. used healthcare records of 1.6 million CYP across two US databases to compare diabetes incidence more than 30 days after COVID‐19 with CYP who were coded as having other acute respiratory tract infections and non‐COVID‐19 events.^25^ They found strong associations between COVID‐19 and new‐onset diabetes, but did not distinguish between types of diabetes. Kendall et al.^26^ examined electronic health records of 571,256 CYP in the United States from the start of the pandemic to December 2021. Similar to our findings, they found around a twofold increase in hazard of type 1 diabetes at 1, 3 and 6 months of follow‐up among CYP with SARS‐CoV‐2 infection compared with matched cohorts presenting with other respiratory tract infections and CYP presenting with fractures or for well child visits.^26^ A recent metanalysis using data from seven studies found around a 40% increase in risk of type 1 diabetes within CYP exposed to SARS‐CoV‐2, although there was a high degree of heterogeneity in included studies.^28^

Our findings differ from McKeigue et al., who found no association between SARS‐CoV‐2 infection and type 1 diabetes in a study using data on more than 350,000 CYP from the REACT‐SCOT COVID‐19 matched case control study, linked to national SARS‐COV‐2 test and diabetes diagnosis data.^29^ Noorzae et al. also found no association between positive SARS‐CoV‐2 tests and incident type 1 diabetes.^30^ This Danish population cohort used similar methods to our study and analysed data from March 2020 to August 2022. The authors adjusted their analyses for pre‐existing comorbidities using Charlson's comorbidity index, a scale more commonly used in adults and which does not identify CYP with many conditions which predispose to type 1 diabetes. We also found some evidence that associations between SARS‐CoV‐2 exposure and type 1 diabetes may be strongest during Delta, and this period contributed to under 10% of the total person years of follow‐up in Noorzae et al. Overall, we were able to analyse outcomes in a far larger population of CYP than Noorzae et al.^30^ and McKeigue et al.^29^ and excluded all CYP with any prior chronic medical problems from our analysis, which may also explain some of the differences in findings.

### 3.2 Strengths and weaknesses

We used whole population‐level data of all hospitalisations in England, linked with multiple other health datasets including a robust national diabetes audit and a comprehensive national COVID‐19 testing dataset, to construct a cohort of CYP exposed to SARS‐CoV‐2 during the first two years of the pandemic. We then compared type 1 diabetes incidence against four unexposed cohorts, after excluding CYP with any pre‐recorded chronic medical conditions using established code lists. As CYP with newly diagnosed diabetes are routinely hospitalised at the time of diagnosis in England, we are likely to have captured the great majority of CYP with incident diabetes during the study period.

Limitations to this analysis include incomplete or inaccurate diagnostic and sociodemographic coding within SUS, and in linkages with the other datasets used. Although we had access to date and type of diabetes diagnosis within the NDA, we did not have access to prescription or autoantibody data to confirm the accuracy of this, and some cases may be misclassified. Further, type of diabetes was missing in around 9% of CYP diagnosed during follow‐up. Although we were able to control for important sociodemographic characteristics, and remove from the analysis those with previous chronic conditions which may be associated with type 1 diabetes, we were limited in covariates to include, and this may have resulted in residual confounding. Importantly, we were unable to adjust for exposure to infections other than SARS‐CoV‐2, or account for changes in testing capacity and behaviour as the pandemic progressed, which may have affected our results. There was also large disruption in patterns of healthcare‐seeking behaviour during the pandemic, which we were unable to fully account for in our model. There were differences between the examined cohorts in addition to exposure status, with the exposed cohort having larger numbers of White female CYP from less deprived backgrounds than the unexposed cohorts, although we did adjust for these factors in our analysis. This likely reflects variation in SARS‐CoV‐2 testing uptake^37^ and care‐seeking behaviour in certain groups, and we are likely to have missed exposed CYP who did not have a positive test registered. We may have therefore underestimated differences between cohorts, as there are likely to be CYP who were exposed to SARS‐CoV‐2 but never tested within the unexposed cohorts. We relied on hospital admission data to identify cohorts of unexposed CYP during the pandemic which may not be representative of unexposed CYP in the community. Further, there were large differences in outcomes between the unexposed cohorts. The traumatic cohort may have been more representative than the elective cohort; we found very similar estimates for the incidence of type 1 diabetes within the traumatic cohort for CYP aged 0–15 for the year prior to the pandemic to that reported in the National Paediatric Diabetes Audit (24.9 per 100,000 compared with 24.6 per 100,000). We found higher incidence estimates within the elective cohort, which may reflect planned inpatient investigations for undiagnosed conditions related to diabetes within this population. The large increase in type 1 diabetes incidence within 28 days of elective admissions in both the pandemic and historic cohorts may be further evidence of this.

### 3.3 Meaning and mechanisms

Our results support growing evidence of an association between SARS‐CoV‐2 infection and new‐onset type 1 diabetes. The Royal College of Paediatrics and Child Health (RCPCH) National Paediatric Diabetes Audit has highlighted areas where improvements have been made to CYP diabetes management in the UK over recent years, although inequalities remain.^9^ Increased incidence of type 1 diabetes driven by SARS‐CoV‐2 infection may threaten capacity to sustain these improvements, particularly should cases of COVID‐19 increase, or further SARS‐CoV‐2 variants or new future pandemics emerge.

The mechanisms underlying the association we report here are complex and yet to be fully established.^14^ Recent work suggests SARS‐CoV‐2 infection may accelerate clinical diabetes in CYP known to have islet autoantibodies,^15^ although this has not been found in other analyses.^31^ SARS‐CoV‐2 infection may also be associated with developing islet cell autoantibodies in young children.^38^ This raises multiple areas for future study, including further studies within those known to be at risk of developing type 1 diabetes, examining variation by age, analysing possible impacts of SARS‐CoV‐2 infection on T1 diabetes incidence over longer periods, and the role of vaccination in mediating these associations. If further work establishes a causal relationship between SARS‐CoV‐2 and T1DM, public health messaging may need to highlight the importance of recognising symptoms associated with T1DM after SARS‐CoV‐2 infection.

We found evidence that exposure to SARS‐CoV‐2 is associated with incident type 1 diabetes. This suggests SARS‐CoV‐2 infection may contribute to the increases in type 1 diabetes diagnoses in CYP observed in multiple studies in other European countries and the United States,^1^, ^2^, ^3^, ^4^ and potentially in the decline in diagnoses observed as the pandemic subsided.^8^ Our findings have potential implications for future type 1 diabetes disease burden and prompt further work to understand the underlying mechanisms of these associations.

## FUNDING INFORMATION

This study is funded by the NIHR [ref 202322]. The views expressed are those of the authors and not necessarily those of the NIHR or the Department of Health and Social Care.

## CONFLICT OF INTEREST STATEMENT

KK has acted as a consultant, speaker or received grants for investigator‐initiated studies for Astra Zeneca, Bayer, Novartis, Novo Nordisk, Sanofi‐Aventis, Lilly and Merck Sharp & Dohme, Boehringer Ingelheim, Oramed Pharmaceuticals, Pfizer, Roche, Daiichi‐Sankyo and Applied Therapeutics. KK was chair of the ethnicity subgroup of the UK Scientific Advisory Group for Emergencies (SAGE) and a member of SAGE. JV was the National Clinical Director for Diabetes and Obesity at NHS England from April 2013 to September 2023.

## ETHICAL APPROVAL AND LEGAL BASIS FOR DATA LINKAGE AND ANALYSES

Ethics approval was provided after review by Yorkshire and the Humber, South Yorkshire NHS Research Ethics Committee on 10th June 2021 (Reference 21/YH/0127). Current Control Of Patient Information (COPI) regulations provide a legal basis for linking these datasets without consent.^39^

## Supporting information


Data S1.

Data S2.

## Data Availability

These analyses were undertaken using datasets held by NHS England for the use of ongoing service evaluation, held within the National Commissioning Data Repository. Access to these data at individual level are restricted, as described in data sharing agreements between NHS England and specific data providers, and within the application for ethical approval provided for this study.

## References

[1] Unsworth R , Wallace S , Oliver NS , et al. New‐onset type 1 diabetes in children during COVID‐19: multicenter regional findings in the UK. Diabetes Care. 2020;43(11):e170‐e171. 32816997 10.2337/dc20-1551

[2] Kamrath C , Rosenbauer J , Eckert AJ , et al. Incidence of type 1 diabetes in children and adolescents during the COVID‐19 pandemic in Germany: results from the DPV registry. Diabetes Care. 2022;45(8):1762‐1771. 35043145 10.2337/dc21-0969

[3] Rahmati M , Keshvari M , Mirnasuri S , et al. The global impact of COVID‐19 pandemic on the incidence of pediatric new‐onset type 1 diabetes and ketoacidosis: a systematic review and meta‐analysis. J Med Virol. 2022;94(11):5112‐5127. 35831242 10.1002/jmv.27996 PMC9350204

[4] D'Souza D , Empringham J , Pechlivanoglou P , Uleryk EM , Cohen E , Shulman R . Incidence of diabetes in children and adolescents during the COVID‐19 pandemic: a systematic review and meta‐analysis. JAMA Netw Open. 2023;6(6):e2321281. 37389869 10.1001/jamanetworkopen.2023.21281 PMC10314307

[5] Lawrence C , Seckold R , Smart C , et al. Increased paediatric presentations of severe diabetic ketoacidosis in an Australian tertiary centre during the COVID‐19 pandemic. Diabet Med. 2021;38(1):e14417. 33020999 10.1111/dme.14417 PMC7646057

[6] Kamrath C , Mönkemöller K , Biester T , et al. Ketoacidosis in children and adolescents with newly diagnosed type 1 diabetes during the COVID‐19 pandemic in Germany. JAMA. 2020;324(8):801‐804. 32702751 10.1001/jama.2020.13445 PMC7372511

[7] Royal College of Paediatrics and Child Health . National Paediatric Diabetes Audit Annual report 2020–21: care processes and outcomes. 2022.

[8] Berthon W , McGurnaghan SJ , Blackbourn LA , et al. Incidence of type 1 diabetes in children has fallen to pre–COVID‐19 pandemic levels: a population‐wide analysis from Scotland. Diabetes Care. 2024;47(3):e26‐e28. 38113438 10.2337/dc23-2068

[9] Royal College of Paediatrics and Child Health . National Paediatric Diabetes Audit (NPDA) Report on Care and Outcomes 2022/23. 2024.

[10] Filippi CM , von Herrath MG . Viral trigger for type 1 diabetes: pros and cons. Diabetes. 2008;57(11):2863‐2871. 18971433 10.2337/db07-1023 PMC2570378

[11] Laitinen OH , Honkanen H , Pakkanen O , et al. Coxsackievirus B1 is associated with induction of β‐cell autoimmunity that portends type 1 diabetes. Diabetes. 2014;63(2):446‐455. 23974921 10.2337/db13-0619

[12] Op de Beeck A , Eizirik DL . Viral infections in type 1 diabetes mellitus—why the β cells? Nat Rev Endocrinol. 2016;12(5):263‐273. 27020257 10.1038/nrendo.2016.30 PMC5348720

[13] Kondrashova A , Hyöty H . Role of viruses and other microbes in the pathogenesis of type 1 diabetes. Int Rev Immunol. 2014;33(4):284‐295. 24611784 10.3109/08830185.2014.889130

[14] Khunti K , Del Prato S , Mathieu C , Kahn SE , Gabbay RA , Buse JB . COVID‐19, hyperglycemia, and new‐onset diabetes. Diabetes Care. 2021;44(12):2645‐2655. 34625431 10.2337/dc21-1318 PMC8669536

[15] Friedl N , Sporreiter M , Winkler C , et al. Progression from presymptomatic to clinical type 1 diabetes after covid‐19 infection. JAMA. 2024;332(6):501‐502. 39008327 10.1001/jama.2024.11174 PMC11250358

[16] Groß R , Kleger A . COVID‐19 and diabetes—where are we now? Nat Metab. 2022;4(12):1611‐1613. 36369292 10.1038/s42255-022-00691-w

[17] Accili D . Can COVID‐19 cause diabetes? Nat Metab. 2021;3(2):123‐125. 33432203 10.1038/s42255-020-00339-7 PMC8892570

[18] Xie Y , Al‐Aly Z . Risks and burdens of incident diabetes in long COVID: a cohort study. Lancet Diabetes Endocrinol. 2022;10(5):311‐321. 35325624 10.1016/S2213-8587(22)00044-4 PMC8937253

[19] Zhang T , Mei Q , Zhang Z , et al. Risk for newly diagnosed diabetes after COVID‐19: a systematic review and meta‐analysis. BMC Med. 2022;20(1):444. 36380329 10.1186/s12916-022-02656-y PMC9666960

[20] Rathmann W , Kuss O , Kostev K . Incidence of newly diagnosed diabetes after Covid‐19. Diabetologia. 2022;65(6):949‐954. 35292829 10.1007/s00125-022-05670-0 PMC8923743

[21] Bull‐Otterson L , Baca S , Saydah S , et al. Post–COVID conditions among adult COVID‐19 survivors aged 18–64 and ≥65 years—United States, March 2020–November 2021. Morb Mortal Wkly Rep. 2022;71(21):713‐717.

[22] Qeadan F , Tingey B , Egbert J , et al. The associations between COVID‐19 diagnosis, type 1 diabetes, and the risk of diabetic ketoacidosis: a nationwide cohort from the US using the Cerner real‐world data. PLoS One. 2022;17(4):e0266809. 35439266 10.1371/journal.pone.0266809 PMC9017888

[23] Chang R , Chen TY‐T , Wang S‐I , Hung Y‐M , Chen H‐Y , Wei C‐CJ . Risk of autoimmune diseases in patients with COVID‐19: a retrospective cohort study. EClinicalMedicine. 2023;56:101783. 36643619 10.1016/j.eclinm.2022.101783 PMC9830133

[24] Tesch F , Ehm F , Vivirito A , et al. Incident autoimmune diseases in association with SARS‐CoV‐2 infection: a matched cohort study. Clin Rheumatol. 2023;42(10):2905‐2914. 37335408 10.1007/s10067-023-06670-0 PMC10497688

[25] Barrett CE , Koyama AK , Alvarez P , et al. Risk for newly diagnosed diabetes >30 days after SARS‐CoV‐2 infection among persons aged <18 years—United States, march 1, 2020–June 28, 2021. Morb Mortal Wkly Rep. 2022;71(2):59‐65. 10.15585/mmwr.mm7102e2 PMC8757617 35025851

[26] Kendall EK , Olaker VR , Kaelber DC , Xu R , Davis PB . Association of SARS‐CoV‐2 infection with new‐onset type 1 diabetes among pediatric patients from 2020 to 2021. JAMA Netw Open. 2022;5(9):e2233014. 36149658 10.1001/jamanetworkopen.2022.33014 PMC9508649

[27] Kompaniyets L . Post–COVID‐19 symptoms and conditions among children and adolescents—United States, March 1, 2020–January 31, 2022. MMWR Morb Mortal Wkly Rep. 2022;71:999. 10.15585/mmwr.mm7131a3 PMC9368731 35925799

[28] Rahmati M , Yon DK , Lee SW , et al. New‐onset type 1 diabetes in children and adolescents as postacute sequelae of SARS‐CoV‐2 infection: a systematic review and meta‐analysis of cohort studies. J Med Virol. 2023;95(6):e28833. 37264687 10.1002/jmv.28833

[29] McKeigue PM , McGurnaghan S , Blackbourn L , et al. Relation of incident type 1 diabetes to recent COVID‐19 infection: cohort study using e‐health record linkage in Scotland. Diabetes Care. 2023;46(5):921‐928. 35880797 10.2337/dc22-0385

[30] Noorzae R , Junker TG , Hviid AP , Wohlfahrt J , Olsen SF . Risk of type 1 diabetes in children is not increased after SARS‐CoV‐2 infection: a nationwide prospective study in Denmark. Diabetes Care. 2023;46(6):1261‐1264. 37058353 10.2337/dc22-2351

[31] Krischer JP , Lernmark Å , Hagopian WA , et al. SARS‐CoV‐2—no increased islet autoimmunity or type 1 diabetes in teens. N Engl J Med. 2023;389(5):474‐475. 37530831 10.1056/NEJMc2216477 PMC10481371

[32] Bering L , Christensen AV , Birk NM , et al. Risk of new‐onset type 1 diabetes in Danish children and adolescents after SARS‐CoV‐2 infection: a Nationwide, matched cohort study. Pediatr Infect Dis J. 2023;42(11):999‐1001. 37566892 10.1097/INF.0000000000004063

[33] Usher‐Smith JA , Thompson MJ , Zhu H , Sharp SJ , Walter FM . The pathway to diagnosis of type 1 diabetes in children: a questionnaire study. BMJ Open. 2015;5(3):e006470. 10.1136/bmjopen-2014-006470 PMC4368911 25783422

[34] Holman N , Knighton P , Wild SH , et al. Cohort profile: national diabetes audit for England and Wales. Diabet Med. 2021;38(9):e14616. 34062007 10.1111/dme.14616

[35] Office for National Statistics . Coronavirus (COVID‐19) latest insights: Infections. 2023.

[36] Thomas R . All hospital emergency patients to be tested for coronavirus. Health Serv J. 2020. Accessed March 1, 2023. https://www.hsj.co.uk/coronavirus/all‐hospital‐emergency‐patients‐to‐be‐tested‐for‐coronavirus/7027526.article

[37] Green MA , García‐Fiñana M , Barr B , et al. Evaluating social and spatial inequalities of large scale rapid lateral flow SARS‐CoV‐2 antigen testing in COVID‐19 management: an observational study of Liverpool, UK (November 2020 to January 2021). Lancet Regional Health–Europe. 2021;6. 10.1016/j.lanepe.2021.100107 PMC8114854 34002172

[38] Lugar M , Eugster A , Achenbach P , et al. SARS‐CoV‐2 infection and development of islet autoimmunity in early childhood. JAMA. 2023;330(12):1151‐1160. 37682551 10.1001/jama.2023.16348 PMC10523173

[39] NHS Digital . Control of patient information (COPI) notice. 2021. Accessed June 1, 2021. https://digital.nhs.uk/coronavirus/coronavirus‐covid‐19‐response‐information‐governance‐hub/control‐of‐patient‐information‐copi‐notice

