# Supplementary material for: SARS‐CoV‐2 infection and new‐onset type 1 diabetes in the post‐acute period among children and young people in England
Source: Diabet Med. 2025 Jun 17;42(9):e70084. doi: 10.1111/dme.70084 (PMC12352718; doi:10.1111/dme.70084)
Supplement: Supplementary file 1 — Data S1. [file DME-42-e70084-s001.docx]

STROBE Statement—checklist of items that should be included in reports of observational studies

|  | Item No. | Recommendation | Page  No. | Relevant text from manuscript |
| --- | --- | --- | --- | --- |
| **Title and abstract** | 1 | (*a*) Indicate the study’s design with a commonly used term in the title or the abstract | 2 | “We undertook a cohort study” |
|  |  | (*b*) Provide in the abstract an informative and balanced summary of what was done and what was found | 2 |  |
| Introduction | | | |  |
| Background/rationale | 2 | Explain the scientific background and rationale for the investigation being reported | 5-6 | “Although there is clinical and laboratory evidence to suggest SARS-CoV-2 infection may itself trigger diabetes, the mechanisms for this are not fully understood.^14-16^ Some epidemiological studies have reported increases in new onset diabetes after SARS-CoV-2 infection both in the acute and post-acute period in adults.^17-23^ Studies examining this association in CYP have been mixed, with data from the U.S Centers for Disease Control and Prevention (CDC)^24^ and others^252126^*^27^* finding an increase in T1DM incidence 30 days after SARS-CoV-2 infection, but other work finding no association.^28-31^ Establishing if SARS-CoV-2 infection is associated with an increased risk of developing T1DM is important as this may have consequences for future disease burden and prevention strategies in CYP.” |
| Objectives | 3 | State specific objectives, including any prespecified hypotheses | 5 | “Here we use a national dataset containing all hospital activity in England from 2015, linked to SARS-CoV-2 testing data and date and type of all diabetes diagnoses in CYP. In this population cohort study, we use these unique linked datasets to investigate the association between post-acute SARS-CoV-2 infection and new onset T1DM in CYP in England during the first two years of the pandemic.” |
| Methods | | | |  |
| Study design | 4 | Present key elements of study design early in the paper | 7 | See sections on : Data, population, exposed cohort, unexposed cohort, exclusions, outcomes, follow up, covariates, analysis |
| Setting | 5 | Describe the setting, locations, and relevant dates, including periods of recruitment, exposure, follow-up, and data collection | 7 | See sections on : Data, population, exposed cohort, unexposed cohort, exclusions, outcomes, follow up, covariates, analysis |
| Participants | 6 | (*a*) *Cohort study*—Give the eligibility criteria, and the sources and methods of selection of participants. Describe methods of follow-up  *Case-control study*—Give the eligibility criteria, and the sources and methods of case ascertainment and control selection. Give the rationale for the choice of cases and controls  *Cross-sectional study*—Give the eligibility criteria, and the sources and methods of selection of participants | 7 | CYP aged 0-17 were eligible for inclusion in this study if they had been admitted to hospital in England for any reason at any time from 1^st^ March 2015 to 31^st^ August 2022 |
|  |  | (*b*) *Cohort study*—For matched studies, give matching criteria and number of exposed and unexposed  *Case-control study*—For matched studies, give matching criteria and the number of controls per case |  |  |
| Variables | 7 | Clearly define all outcomes, exposures, predictors, potential confounders, and effect modifiers. Give diagnostic criteria, if applicable | 7 | See sections on : Data, population, exposed cohort, unexposed cohort, exclusions, outcomes, follow up, covariates, analysis |
| Data sources/ measurement | 8* | For each variable of interest, give sources of data and details of methods of assessment (measurement). Describe comparability of assessment methods if there is more than one group | 7 | We used Secondary Use Services (SUS) data, containing sociodemographic characteristics and clinical details of all individuals admitted to hospital from March 2015 to August 2022. SUS data were deterministically linked to: 1) the National Diabetes Audit^33^ (NDA), providing type and date of diabetes diagnosis; 2) SARS-CoV-2 positive test data held by NHS England (date of positive polymerase chain reaction and lateral flow tests in the community and in hospital); 3) mortality data from the Office for National Statistics and National Child Mortality Database. |
| Bias | 9 | Describe any efforts to address potential sources of bias |  | To compare incidence of T1DM in otherwise healthy CYP, we identified and excluded all those with any chronic medical problems recorded within SUS prior to each cohort index date, using established code lists (see supplementary table S1) |
| Study size | 10 | Explain how the study size was arrived at |  | The study size was determined by the total number of CYP who had been admitted to hospital between 2015 and 2022 and had either; a positive SARS-CoV-2 test or and admission due to trauma or for an elective procedure |

Continued on next page

| Quantitative variables | 11 | Explain how quantitative variables were handled in the analyses. If applicable, describe which groupings were chosen and why | 9 | See section below |
| --- | --- | --- | --- | --- |
| Statistical methods | 12 | (*a*) Describe all statistical methods, including those used to control for confounding | 9 | We first describe the incidence rate of T1DM during follow up amongst CYP exposed to SARS-CoV-2 and those not exposed from day 0 to day 27 (acute period), day 28-day 209 (post-acute period), and from day 210 onwards (late period). We estimated Kaplan-Meier survival curves to describe the probability of developing T1DM during the post-acute follow up period by exposure and cohort. We then used Cox regression survival analyses to compare the relative hazard of developing T1DM within CYP exposed and unexposed to SARS-CoV-2, adjusted for age group, sex, ethnicity and IMD quintile, and index date season, separately for each period during the post-acute period. We examined the association between the exposed cohort and each of the unexposed cohorts separately (note CYP could be in both the elective and traumatic unexposed cohorts). We then examined these associations according to the dominant SARS-CoV-2 variant in England at the time CYP entered each cohort. We undertook the analysis in STATA 16, using the command *stcox*. We assessed the assumption of proportional hazards by examining trends in Schoenfeld’s residuals using the command *stphtest*. |
|  |  | (*b*) Describe any methods used to examine subgroups and interactions | 9 | See section above |
|  |  | (*c*) Explain how missing data were addressed | N/A |  |
|  |  | (*d*) *Cohort study*—If applicable, explain how loss to follow-up was addressed  *Case-control study*—If applicable, explain how matching of cases and controls was addressed  *Cross-sectional study*—If applicable, describe analytical methods taking account of sampling strategy | 9 | See section above |
|  |  | (*e*) Describe any sensitivity analyses | N/A |  |
| Results | | | | |
| Participants | 13* | (a) Report numbers of individuals at each stage of study—eg numbers potentially eligible, examined for eligibility, confirmed eligible, included in the study, completing follow-up, and analysed | 11 | We identified 1,087,604 CYP in the exposed cohort, 143,748 in the trauma cohort, 253,368 in the elective cohort, 160,925 in the historic trauma cohort and 388,673 in the historic elective cohort **(Table 1)**. |
|  |  | (b) Give reasons for non-participation at each stage | N/A |  |
|  |  | (c) Consider use of a flow diagram |  |  |
| Descriptive data | 14* | (a) Give characteristics of study participants (eg demographic, clinical, social) and information on exposures and potential confounders |  | See Table 1 |
|  |  | (b) Indicate number of participants with missing data for each variable of interest |  | See Table 1 |
|  |  | (c) *Cohort study*—Summarise follow-up time (eg, average and total amount) |  |  |
| Outcome data | 15* | *Cohort study*—Report numbers of outcome events or summary measures over time |  | See Table 2 and figure 1 |
|  |  | *Case-control study—*Report numbers in each exposure category, or summary measures of exposure |  |  |
|  |  | *Cross-sectional study—*Report numbers of outcome events or summary measures |  |  |
| Main results | 16 | (*a*) Give unadjusted estimates and, if applicable, confounder-adjusted estimates and their precision (eg, 95% confidence interval). Make clear which confounders were adjusted for and why they were included |  | See Table 3, supplementary tables 8 and 9 and figure S6 |
|  |  | (*b*) Report category boundaries when continuous variables were categorized |  |  |
|  |  | (*c*) If relevant, consider translating estimates of relative risk into absolute risk for a meaningful time period |  |  |

Continued on next page

| Other analyses | 17 | Report other analyses done—eg analyses of subgroups and interactions, and sensitivity analyses |  |  |
| --- | --- | --- | --- | --- |
| Discussion | | | | |
| Key results | 18 | Summarise key results with reference to study objectives | 13 | In this population cohort study of previously well CYP in England, we found exposure to SARS-CoV-2 was associated with significantly higher incidence of developing T1DM compared with CYP who were not exposed. |
| Limitations | 19 | Discuss limitations of the study, taking into account sources of potential bias or imprecision. Discuss both direction and magnitude of any potential bias | 14 | See strengths and limitations |
| Interpretation | 20 | Give a cautious overall interpretation of results considering objectives, limitations, multiplicity of analyses, results from similar studies, and other relevant evidence | 15 | See meaning and mechanisms |
| Generalisability | 21 | Discuss the generalisability (external validity) of the study results | 15 | See conclusion |
| Other information | |  | | |
| Funding | 22 | Give the source of funding and the role of the funders for the present study and, if applicable, for the original study on which the present article is based | 17 | This study is funded by the NIHR [ref 202322]. The views expressed are those of the authors and not necessarily those of the NIHR or the Department of Health and Social Care. |

*Give information separately for cases and controls in case-control studies and, if applicable, for exposed and unexposed groups in cohort and cross-sectional studies.

**Note:** An Explanation and Elaboration article discusses each checklist item and gives methodological background and published examples of transparent reporting. The STROBE checklist is best used in conjunction with this article (freely available on the Web sites of PLoS Medicine at http://www.plosmedicine.org/, Annals of Internal Medicine at http://www.annals.org/, and Epidemiology at http://www.epidem.com/). Information on the STROBE Initiative is available at www.strobe-statement.org.
