# Supplementary material for: SARS‐CoV‐2 infection and new‐onset type 1 diabetes in the post‐acute period among children and young people in England
Source: Diabet Med. 2025 Jun 17;42(9):e70084. doi: 10.1111/dme.70084 (PMC12352718; doi:10.1111/dme.70084)
Supplement: Supplementary file 2 — Data S2. [file DME-42-e70084-s002.docx]

## Post-acute SARS-CoV-2 infection and new onset type 1 diabetes amongst Children and Young People in England

Supplementary Material

[**Table S1** List of conditions used to exclude CYP with previous medical problems from the analysis 3](#_Toc177130418)

[**Table S2** Leading reasons for previous admission resulting in inclusion within the SUS dataset: elective cohort 9](#_Toc177130419)

[**Table S3** Leading reasons for previous admission resulting in inclusion within the SUS dataset: elective historic cohort 10](#_Toc177130420)

[**Table S4** Leading reasons for previous admission resulting in inclusion within the SUS dataset: traumatic cohort 11](#_Toc177130421)

[**Table S5** Leading reasons for previous admission resulting in inclusion within the SUS dataset: traumatic historic cohort 12](#_Toc177130422)

[**Table S6** Leading reasons for previous admission resulting in inclusion within the SUS dataset: exposed cohort 13](#_Toc177130423)

[**Table S7** Number and percentage of CYP who developed new onset type 1 diabetes within each cohort during follow up 14](#_Toc177130424)

[**Table S8** Number and percentage of CYP who developed new onset type 1 diabetes when different SARS-CoV-2 variants were dominant 15](#_Toc177130425)

[**Table S9** Incidence rate of new onset type 1 diabetes per 100,000 person-years of observation when different SARS-CoV-2 variants were dominant 16](#_Toc177130426)

[**Table S10** Hazard ratio of developing new onset diabetes from day 28 to day 208 in the exposed cohort compared with each unexposed cohort when different SARS-CoV-2 variants were dominant 18](#_Toc177130427)

[**Figure S1** Number of emergency admissions due to a traumatic cause by month in 0-17 year olds in England 2017 - 2023 19](#_Toc198305317)

[**Figure S2** Number of elective admissions for any cause by month in 0-17 year olds in England 2017 - 2023 20](#_Toc198305318)

[**Figure S3** Number of new diagnoses of T1DM within SUS by month in 0-17 year olds in England 2017 - 2023 21](#_Toc198305319)

[**Figure S4** Number of CYP with first positive test for SARS-CoV-2 within SUS by month in 0-17 year olds in England 22](#_Toc198305320)

[**Figure S5** Survival graphs showing probability of new diagnosis of type 1 diabetes in exposed compared with unexposed cohorts 23](#_Toc198305321)

[**Figure S6** Incidence rate of new onset type 1 diabetes from day 28 to 208 days of follow up per 100,000 person-years of observation by dominant variant 26](#_Toc198305322)

**Table S1** List of conditions used to exclude CYP with previous medical problems from the analysis

| Type of chronic condition | Categories | Codes |
| --- | --- | --- |
| Mental health/behavioural | Substance abuse | E24.4, F10-F19, F55, G24.0, G31.2, G40.5, G62.1, G72.0, G72.1, I42.6, K29.2, K70,K85.2, K85.3, K86.0, O35.4, R78.1-R78.5, Y47, Y49, Z50.2, Z50.3, Z71.4, Z71.5,Z72.2, Z86.4 |
|  | Self-harm | X60-X84, Y10-Y34†, Y87.0, Y87.2†, Z91.5 |
|  | Other mental health problems | F00-F01, F02.8, F03-F09, F20-F48, F50, F53, F54, F59, F60-F69, F99, Z09.3,Z50.4, Z86.5, Z91.4 |
|  | Behavioural/developmental disorders | F70-F79, F80.0-F80.2, F80.8, F80.9, F81-F84, F88, F89, F90-F98 |
| Cancer/blood disorders | Neoplasms | C00-C97, D00-D02, D05-D09, D12, D13, D14.1-D14.4, D15, D20, D32-D35, D37-D48, D63.0, E34.0, E88.3, G13.0, G13.1, G53.3, G55.0, G63.1, G73.1, G73.2, G94.1,M36.0, M36.1, M49.5, M82.0, M90.6, M90.7, N08.1, N16.1, Y43.1-Y43.3, Y84.2, Z08,Z51.0-Z51.2, Z54.1, Z54.2, Z85, Z86.0, Z92.3 |
|  | Immunological disorders | D80-D84, G53.2, Q98.0 |
|  | Anaemia and other blood disorders | D50, D56.0-D56.2, D56.4, D56.8, D56.9, D57.0-D57.2, D57.8, D58, D61.0, D61.9,D64, D66, D67, D68.0-D68.2, D68.4-D68.9, D69, D70-D76, M36.2-M36.4, M90.4,N08.2, Z86.2 |
| Chronic infections | HIV | B20-B24, F02.4, R75, Z21 |
|  | Tuberculosis | A15-A19, E35.0, K23.0, K67.3, K93.0, M01.1, M49.0, P37.0 |
|  | Other | A50, A81, B18, B37.1, B37.5, B37.6, B37.7, B38.1, B39.1, B40.1, B44.0, B44.7, B45,B46, B48.7, B50.0, B50.8, B51.0, B51.8, B52.8, B52.0, B55, B57.2-B57.5, B58.0,B59, B67, B69, B73, B74, B78.7, B90-B94, F02.1, K23.1, K93.1, M00, N33.0, P35.0-P35.2, P35.8, P35.9, P37.1 |
| Respiratory | Asthma and chronic lower respiratory disease | J41-J47 |
|  | Cystic fibrosis | E84, P75 |
|  | Injuries | S17, S27, S28, T27, T91.4 |
|  | Congenital anomalies | Q30-Q37, Q79.0 |
|  | Other | G47.3, J60-J70, J80-J86, J96.1, J98, P27, Y55.6, Z43.0, Z93.0, Z94.2 |
| Metabolic/endocrine/digestive/renal/genitourinary  Metabolic/endocrine/digestive/renal/genitourinary | Diabetes | E10-E14, G59.0, G63.2, I79.2, M14.2, N08.3, O24, Y42.3 |
|  | Other endocrine | E00, E03.0, E03.1, E07.1, E22.0, E23.0, E25, E26.8, E29.1, E31, E34.1, E34.2, E34.5,E34.8, G13.2, G73.5, Y42.1 |
|  | Metabolic | D55, E70-E72, E74-E78, E79.1-E79.9,E80.0-E80.3, E80.5, E80.7, E83, E85, E88.0,E88.1, E88.2, E88.8, E88.9, G73.6, L99.0, M14.4, M14.3, N16.3 |
|  | Digestive | K20, K21.0, K22, K23.8, K25-K28, K29.0, K29.1, K29.3-K29.9, K31, K50-K52, K55,K57, K59.2, K63.0-K63.3, K66, K72-K76, K80-K83, K85.0, K85.1, K85.8, K85.9,K86.1-K86.9, K87.0, K90, M07.4, M07.5, M09.1, M09.2, T86.4, Z43.2-Z43.4, Z46.5,Z90.3, Z90.4, Z93.2-Z93.5 |
|  | Renal/GU | D63.8, G63.8, G99.8, I68.8, M90.8, N08.4, N00-N05, N07, N11-N15, N16.0, N16.2,N16.4, N16.5, N16.8, N18, N19, N20-N23, N25, N26, N28, N29, N31, N32, N33.8,N35, N36, N39.1, N39.3, N39.4, N40-N42, N70-N74, N80-N82, N85, N86, N87,N88,P96.0, T82.4, T83.1, T83.2, T83.4-T83.9, T85.5, T86.1, Y60.2, Y61.2, Y62.2, Y84.1,Z49, Z93.6, Z94.0, Z99.2 |
|  | Congenital anomalies of the digestive/renal/GU system | Q38.0, Q38.3, Q38.4, Q38.6-Q38.8, Q39, Q40.2, Q40.3, Q40.8, Q40.9, Q41, Q42,Q43.1, Q43.3-Q43.7, Q43.9, Q44, Q45, Q50.0, Q51, Q52.0-Q52.2, Q52.4, Q54.0-Q54.3, Q54.8, Q54.9, Q55.0, Q55.5, Q56, Q60.1, Q60.2, Q60.4-Q60.6, Q61, Q62.0-Q62.6, Q62.8, Q63.0-Q63.2, Q63.8, Q63.9, Q64, Q79.2-Q79.5, Q87.8, Q89.1, Q89.2 |
|  | Injuries | S36, S37, S38, S39.6, S39.7, T06.5, T28, T91.5 |
|  | Other/unspecific | E66, G63.3, G99.0, M14.5, N92, Z86.3, Z93.8 |
| Musculoskeletal/skin  Musculoskeletal/skin | Musculoskeletal/connective tissue | G55.1-G55.3, G63.5, G63.6, G73.7, J99.0, J99.1, L62.0, M05, M06, M07.0-M07.3,M07.6, M08, M09.8, M10-M13, M14.0, M14.6, M14.8, M30-M35, M40-M43, M45-M48,M50-M54, M60-M62, M63.8, M80.1-M80.9, M81.1-M81.9, M82.1, M82.8,M84.0-M84.2, M84.8, M84.9, M85, M86.3-M86.6, M89, M90.0, M91-M94, N08.5,Y45.4 |
|  | Skeletal injuries/amputations | S13, S22.0-S22.2, S22.5, S23, S32, S33, S68.3, S68.4, S68.8, S77, S78,S87, S88, S97, S98.0, S98.2-S98.4, T02, T04, T05, T20.3, T20.7, T21.3,T21.7, T22.3, T22.7, T23.2, T23.3, T23.6, T23.7, T24.3, T24.7, T25.2,T25.3, T25.6, T25.7, T29.3, T29.7, T30.3, T30.7, T31.2-T31.9, T32.2-T32.9, T87.3-T87.6, T91.2 T91.8, T92.6, T93.1, T93.4, T93.6, T94.0, T94.1,T95.0, T95.1, T95.4, T95.8, T95.9, Y83.5, Z89.1, Z89.2, Z89.5-Z89.8, Z97.1 |
|  | Chronic skin disorders | L10, L11.0, L11.8, L11.9, L12-L14, L28, L40-L45, L57, L58.1, L59, L87, L88, L90,L92, L95, L93, L98.5, M09.0, Q80, Q81, Q87.0-Q87.5, Q89.4 |
|  | Congenital anomalies | Q18.8, Q65.0-Q65.2, Q65.8, Q65.9, Q67.5, Q68.2, Q68.3-Q68.5, Q71-Q73, Q74,Q75.3-Q75.9, Q76.1-Q76.4, Q77, Q78, Q79.6, Q79.8, Q82.0-Q82.4, Q82.9, Q86.2,Q89.7-Q89.9 |
| Neurological  Neurological | Epilepsy | F80.3, G40.0-G40.4, G40.6-G40.9, G41, R56.8, Y46.0-Y46.6 |
|  | Cerebral palsy | G80-G83 |
|  | Injuries of brain, nerves, eyes or ears | S05-S08, S12, S14, S24, S34, S44, S54, S64, S74, S84, S94, T06.0-T06.2, T26, T90.4, T90.5, T91.1, T91.3, T92.4, |
|  | Chronic eye conditions | H05.1-H05.9, H13.3, H17, H18, H19.3, H19.8, H21, H26, H27, H28.0-H28.2, H31,H32.8, H33, H34, H35, H40, H42.0, H43, H44, H47, H54.0- H54.2, H54.4, T85.2,T85.3, Z44.2 |
|  | Chronic ear conditions | H60.2, H65.2-H65.4, H66.1-H66.3, H69.0, H70.1, H73.1, H74.0-H74.3, H75.0, H80,H81.0, H81.4, H83.0, H83.2, H90.0, H90.3, H90.5, H90.6, H91, Z45.3 |
|  | Perinatal conditions | P10, P21.0, P52, P57, P90, P91.1, P91.2, P91.6 |
|  | Congenital anomalies of neurological or sensory systems | Q00-Q07, Q10.4, Q10.7, Q11-Q12, Q13.0-Q13.4, Q13.8, Q13.9, Q14-Q16, Q75.0,Q75.1, Q85, Q86.0, Q86.1, Q86.8, Q90-Q93, Q95.2, Q95.3, Q97, Q99 |
|  | Other | F02.2, F02.3,G00-G09, G10-G12, G13.8, G14, G20-G23, G24.1-G24.9, G25-G30,G31.0-G31.1, G31.8, G31.9, G32-G37, G43-G46, G47.0-G47.2, G47.4-G47.9, G50-G52, G53.0, G53.1, G53.8, G54, G55.8, G56-G58, G59.8, G60, G61, G62.0, G62.2-G62.9, G64, G70, G71,G72.2-G72.9, G73.0, G73.3, G90-G93, G94.2, G94.8, G95,G96, G98, G99.1, G99.2, I60-I67, I68.0, I68.2, I69, I72.0, I72.5, T85.0, T85.1, Y46.7-Y46.8, Z98.2 |
| Cardiovascular | Congenital heart disease | Q20-Q26, Q89.3 |
|  | Other | I00-I28, I31-I39, I41, I42.0-I42.5, I42.7-I42.9, I43.0, I43.1, I43.2-I43.8,I44.1-I44.7, I45.1-I45.9, I46-I51, I52.8, I70-I71, I72.1-I72.4, I72.8,I72.9, I73-I77, I79.0, I79.1, I79.8, I81-I82, I98-I99, M03.6, N08.8, Q27,Q28, S26, T82.0-T82.3, T82.5-T82.9, T86.2, Y60.5, Y61.5, Y62.5, Y84.0, Z45.0,Z50.0, Z94.1, Z95 |
| Codes indicating non-specific chronic | - | R62, R63.3, Z43.1, Z51.5, Z75.5, Z93.1, Z99.3 |
|  |  |  |
| †Codes used with age criteria: Age at death (if from death certificates) or age at admission (if from hospital records) must be 10 years or older. |  |  |

Adapted from Hardelid P, Dattani N, Gilbert R. Estimating the prevalence of chronic conditions in children who die in England, Scotland and Wales: a data linkage cohort study. *BMJ open* 2014

**Table S2** Leading reasons for previous admission resulting in inclusion within the SUS dataset: elective cohort

| ICD-10 shorted code | Description | count | % | cumulative proportion |
| --- | --- | --- | --- | --- |
| K0 | Disorders of tooth development and eruption | 46683 | 18.42 | 18.4 |
| Z0 | General examination and investigation | 14590 | 5.76 | 24.2 |
| J3 | Other diseases of upper respiratory tract | 9271 | 3.66 | 27.8 |
| N4 | Diseases of male genital organs | 8108 | 3.20 | 31.0 |
| Q5 | Congenital malformations of genital organs | 7453 | 2.94 | 34.0 |
| J0 | Acute upper respiratory infections | 6722 | 2.65 | 36.6 |
| K4 | Hernia | 6555 | 2.59 | 39.2 |
| G4 | Episodic and paroxysmal disorders | 6468 | 2.55 | 41.8 |
| H5 | strabismus | 5909 | 2.33 | 44.1 |
| S6 | Injuries to the wrist and hand | 5457 | 2.15 | 46.3 |
| P5 | haematological disorders of fetus and newborn | 5153 | 2.03 | 48.3 |
| S0 | Injuries to the head | 5145 | 2.03 | 50.3 |

**Table S3** Leading reasons for previous admission resulting in inclusion within the SUS dataset: elective historic cohort

| ICD-10 shorted code | Description | count | % | cumulative proportion |
| --- | --- | --- | --- | --- |
| K0 | Disorders of tooth development and eruption | 78246 | 20.13 | 20.13 |
| J3 | Other diseases of upper respiratory tract | 20454 | 5.26 | 25.4 |
| H6 | Diseases of external ear | 20295 | 5.22 | 30.6 |
| Z0 | General examination and investigation | 15822 | 4.07 | 34.7 |
| J0 | Acute upper respiratory infections | 15797 | 4.06 | 38.8 |
| N4 | Diseases of male genital organs | 12148 | 3.13 | 41.9 |
| G4 | Episodic and paroxysmal disorders | 11875 | 3.06 | 44.9 |
| Q5 | Congenital malformations of genital organs | 8846 | 2.28 | 47.2 |
| H5 | Strabismus | 7755 | 2.00 | 49.2 |
| M2 | Other joint disorders | 7500 | 1.93 | 51.1 |

**Table S4** Leading reasons for previous admission resulting in inclusion within the SUS dataset: traumatic cohort

| ICD-10 code | Description | count | % | cumulative proportion |
| --- | --- | --- | --- | --- |
| S52 | Fracture of forearm | 16869 | 11.7 | 11.7 |
| S01 | Open wound of head | 13639 | 9.5 | 21.2 |
| T39 | Poisoning by nonopioid analgesics, antipyretics and antirheumatics | 11653 | 8.1 | 29.3 |
| S00 | Superficial injury of head | 7885 | 5.5 | 34.8 |
| S42 | Fracture of shoulder and upper arm | 7850 | 5.5 | 40.3 |
| S61 | Open wound of wrist and hand | 7110 | 4.9 | 45.2 |
| S82 | Fracture of lower leg, including ankle | 6336 | 4.4 | 49.6 |
| T78 | Adverse effects, not elsewhere classified | 5010 | 3.5 | 53.1 |

**Table S5** Leading reasons for previous admission resulting in inclusion within the SUS dataset: traumatic historic cohort

| ICD-10 code | Description | count | % | cumulative proportion |
| --- | --- | --- | --- | --- |
| S52 | Fracture of forearm | 18192 | 11.3 | 11.3 |
| S01 | Open wound of head | 13259 | 8.2 | 19.5 |
| T39 | Poisoning by nonopioid analgesics, antipyretics and antirheumatics | 12360 | 7.7 | 27.2 |
| S00 | Superficial injury of head | 8460 | 5.3 | 32.5 |
| S61 | Open wound of wrist and hand | 8106 | 5.0 | 37.5 |
| S42 | Fracture of shoulder and upper arm | 7576 | 4.7 | 42.2 |
| S82 | Fracture of lower leg, including ankle | 6854 | 4.3 | 46.5 |
| S09 | Other and unspecified injuries of head | 6292 | 3.9 | 50.4 |

**Table S6** Leading reasons for previous admission resulting in inclusion within the SUS dataset: exposed cohort

| ICD-10 code | Description | count | % | cumulative proportion |
| --- | --- | --- | --- | --- |
| Z38 | Liveborn infants according to place of birth | 171256 | 15.7 | 15.7 |
| B34 | Viral infection of unspecified site | 52419 | 4.8 | 20.6 |
| K02 | Dental caries | 42004 | 3.9 | 24.4 |
| J03 | Acute tonsillitis | 38946 | 3.6 | 28.0 |
| R10 | Abdominal and pelvic pain | 34444 | 3.2 | 31.2 |
| J06 | Acute upper respiratory infections of multiple and unspecified sites | 26502 | 2.4 | 33.6 |
| S52 | Fracture of forearm | 22302 | 2.1 | 35.7 |
| J22 | Unspecified acute lower respiratory infection | 17453 | 1.6 | 37.3 |
| P59 | Neonatal jaundice from other and unspecified causes | 15996 | 1.5 | 38.7 |
| A08 | Viral and other specified intestinal infections | 15457 | 1.4 | 40.2 |
| Z03 | Medical observation and evaluation for suspected diseases and conditions | 14621 | 1.3 | 41.5 |
| J21 | Acute bronchiolitis | 13701 | 1.3 | 42.8 |
| S01 | Open wound of head | 13045 | 1.2 | 44.0 |
| A09 | Other gastroenteritis and colitis of infectious and unspecified origin | 12988 | 1.2 | 45.2 |
| K35 | Acute appendicitis | 12513 | 1.2 | 46.3 |
| P07 | Disorders related to short gestation and low birth weight, not classified | 12270 | 1.1 | 47.4 |
| K01 | Embedded and impacted teeth | 10581 | 1.0 | 48.4 |
| N47 | Redundant prepuce, phimosis and paraphimosis | 10503 | 1.0 | 49.4 |
| P20 | Intrauterine hypoxia | 10353 | 1.0 | 50.3 |

**Table S7** Number and percentage of CYP who developed new onset type 1 diabetes within each cohort during follow up

|  | Cohort | Total CYP in cohort | Number of CYP with new onset T1 Diabetes | | | | | | | |
| --- | --- | --- | --- | --- | --- | --- | --- | --- | --- | --- |
|  |  |  | At any time | | less than 28 days | | 28 days to 208 days | | longer than 208 days | |
|  |  |  | n | % | n | % | n | % | n | % |
| Pandemic period | exposed to SARS-CoV-2 | 1,087,604 | 944 | 0.09 | 220 | 0.02 | 475 | 0.04 | 249 | 0.02 |
|  | unexposed traumatic admissions | 143,748 | 58 | 0.04 | 3 | 0.00 | 24 | 0.02 | 31 | 0.02 |
|  | unexposed elective admissions | 253,368 | 115 | 0.05 | 46 | 0.02 | 33 | 0.01 | 36 | 0.01 |
| Pre-pandemic period | unexposed traumatic historic | 160,925 | 38 | 0.02 | 3 | 0.00 | 16 | 0.01 | 19 | 0.01 |
|  | unexposed elective historic | 388,673 | 184 | 0.05 | 40 | 0.01 | 65 | 0.02 | 79 | 0.02 |

**Table S8** Number and percentage of CYP who developed new onset type 1 diabetes when different SARS-CoV-2 variants were dominant

| Cohort | Variant | Total CYP | Number of CYP with new onset T1 Diabetes | | | | | | | |
| --- | --- | --- | --- | --- | --- | --- | --- | --- | --- | --- |
|  |  |  | At any time | | less than 28 days | | 28 days to 208 days | | longer than 208 days | |
|  |  |  | n | % | n | % | n | % | n | % |
| exposed to SARS-CoV-2 | Wild type | 40,002 | 75 | 0.19 | 10 | 0.02 | 20 | 0.05 | 45 | 0.11 |
|  | Alpha | 85,748 | 125 | 0.15 | 18 | 0.02 | 27 | 0.03 | 80 | 0.09 |
|  | Delta | 445,728 | 454 | 0.10 | 92 | 0.02 | 256 | 0.06 | 106 | 0.02 |
|  | Omicron | 516,126 | 290 | 0.06 | 100 | 0.02 | 172 | 0.03 | 18 | 0.00 |
| unexposed traumatic admissions | Wild type | 38,439 | 24 | 0.06 | 0 | 0.00 | 6 | 0.02 | 18 | 0.05 |
|  | Alpha | 25,545 | 9 | 0.04 | 0 | 0.00 | 6 | 0.02 | 3 | 0.01 |
|  | Delta | 37,012 | 18 | 0.05 | 2 | 0.01 | 7 | 0.02 | 9 | 0.02 |
|  | Omicron | 42,752 | 7 | 0.02 | 1 | 0.00 | 5 | 0.01 | 1 | 0.00 |
| unexposed elective admissions | Wild type | 51,884 | 36 | 0.07 | 10 | 0.02 | 9 | 0.02 | 17 | 0.03 |
|  | Alpha | 41,111 | 26 | 0.06 | 10 | 0.02 | 10 | 0.02 | 6 | 0.01 |
|  | Delta | 73,064 | 30 | 0.04 | 9 | 0.01 | 10 | 0.01 | 11 | 0.02 |
|  | Omicron | 87,309 | 23 | 0.03 | 17 | 0.02 | 4 | 0.00 | 2 | 0.00 |

**Table S9** Incidence rate of new onset type 1 diabetes per 100,000 person-years of observation when different SARS-CoV-2 variants were dominant

| Cohort |  | At any time | | | less than 28 days | | | 28 days to 208 days | | | longer than 208 days | | |
| --- | --- | --- | --- | --- | --- | --- | --- | --- | --- | --- | --- | --- | --- |
|  |  | Rate | 95CI% |  | Rate | 95CI% |  | Rate | 95CI% | | Rate | 95CI% | |
| Wild type | exposed to SARS-CoV-2 | 101.7 | 81.1 | 127.5 | 314.7 | 169.3 | 584.9 | 99.8 | 64.4 | 154.7 | 88.5 | 66.1 | 118.6 |
|  | unexposed traumatic admissions | 30.7 | 20.6 | 45.8 | 0.0 | - | - | 31.2 | 14.0 | 69.4 | 32.1 | 20.2 | 50.9 |
|  | unexposed elective admissions | 35.2 | 25.4 | 48.9 | 242.7 | 130.6 | 451.1 | 34.6 | 18.0 | 66.6 | 23.5 | 14.6 | 37.7 |
| Alpha | exposed to SARS-CoV-2 | 90.9 | 76.3 | 108.3 | 264.3 | 166.5 | 419.4 | 62.8 | 43.1 | 91.6 | 90.6 | 72.8 | 112.8 |
|  | unexposed traumatic admissions | 23.8 | 12.4 | 45.7 | 0.0 | - | - | 46.9 | 21.1 | 104.3 | 12.9 | 4.2 | 40.1 |
|  | unexposed elective admissions | 42.5 | 29.0 | 62.5 | 306.4 | 164.8 | 569.4 | 48.6 | 26.1 | 90.3 | 16.0 | 7.2 | 35.5 |
| Delta | exposed to SARS-CoV-2 | 111.4 | 101.6 | 122.2 | 259.8 | 211.8 | 318.7 | 114.6 | 101.4 | 129.6 | 70.0 | 57.9 | 84.7 |
|  | unexposed traumatic admissions | 47.4 | 29.9 | 75.2 | 68.0 | 17.0 | 272.1 | 37.8 | 18.0 | 79.2 | 53.8 | 28.0 | 103.3 |
|  | unexposed elective admissions | 40.7 | 28.5 | 58.3 | 155.1 | 80.7 | 298.2 | 27.3 | 14.7 | 50.8 | 34.7 | 19.2 | 62.6 |
| Omicron | exposed to SARS-CoV-2 | 97.2 | 86.6 | 109.0 | 244.2 | 200.8 | 297.1 | 72.1 | 62.1 | 83.7 | 80.6 | 50.8 | 127.9 |
|  | unexposed traumatic admissions | 48.9 | 23.3 | 102.6 | 31.2 | 4.4 | 221.3 | 38.0 | 15.8 | 91.3 | 229.2 | 32.3 | 1627.3 |
|  | unexposed elective admissions | 76.5 | 50.8 | 115.0 | 258.9 | 161.0 | 416.5 | 14.5 | 5.4 | 38.6 | 210.3 | 52.6 | 840.7 |

**Table S10** Hazard ratio of developing new onset diabetes from day 28 to day 208 in the exposed cohort compared with each unexposed cohort when different SARS-CoV-2 variants were dominant

| Cohort |  | Hazard Ratio | 95% CI | | p | phTest |
| --- | --- | --- | --- | --- | --- | --- |
| Unexposed Traumatic |  | 1 |  |  |  |  |
| Exposed | Wild type | 3.1 | 1.00 | 9.85 | 0.05 | 0.84 |
|  | Alpha | 1.1 | 0.44 | 2.97 | 0.80 | 0.18 |
|  | Delta | 2.6 | 1.24 | 5.65 | 0.01 | 0.50 |
|  | Omicron | 1.9 | 0.73 | 4.84 | 0.19 | 0.98 |
| Unexposed Elective |  | 1 |  |  |  |  |
| Exposed | Wild type | 3.2 | 1.26 | 8.01 | 0.01 | 0.54 |
|  | Alpha | 1.2 | 0.53 | 2.60 | 0.69 | 0.24 |
|  | Delta | 3.5 | 1.82 | 6.56 | <0.001 | 0.42 |
|  | Omicron | 4.3 | 1.52 | 11.88 | 0.01 | 0.94 |

*Models adjusted for sex, age, ethnicity, IMD quintile, index date season

**Figure S1** Number of emergency admissions due to a traumatic cause by month in 0-17 year olds in England 2017 - 2023


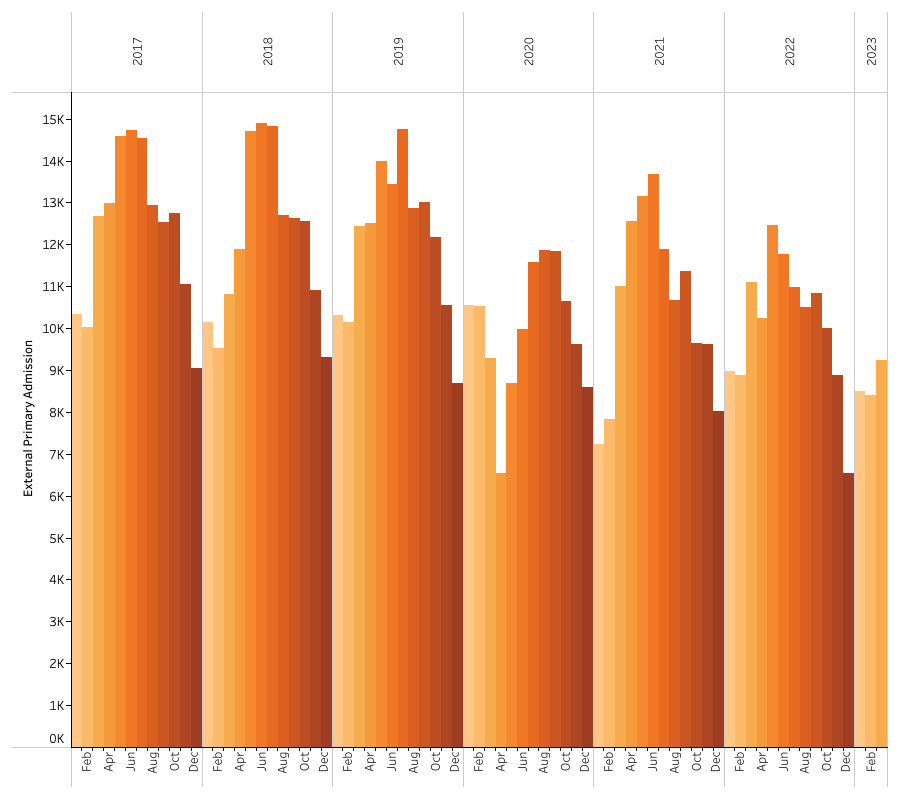


**Figure S2** Number of elective admissions for any cause by month in 0-17 year olds in England 2017 - 2023


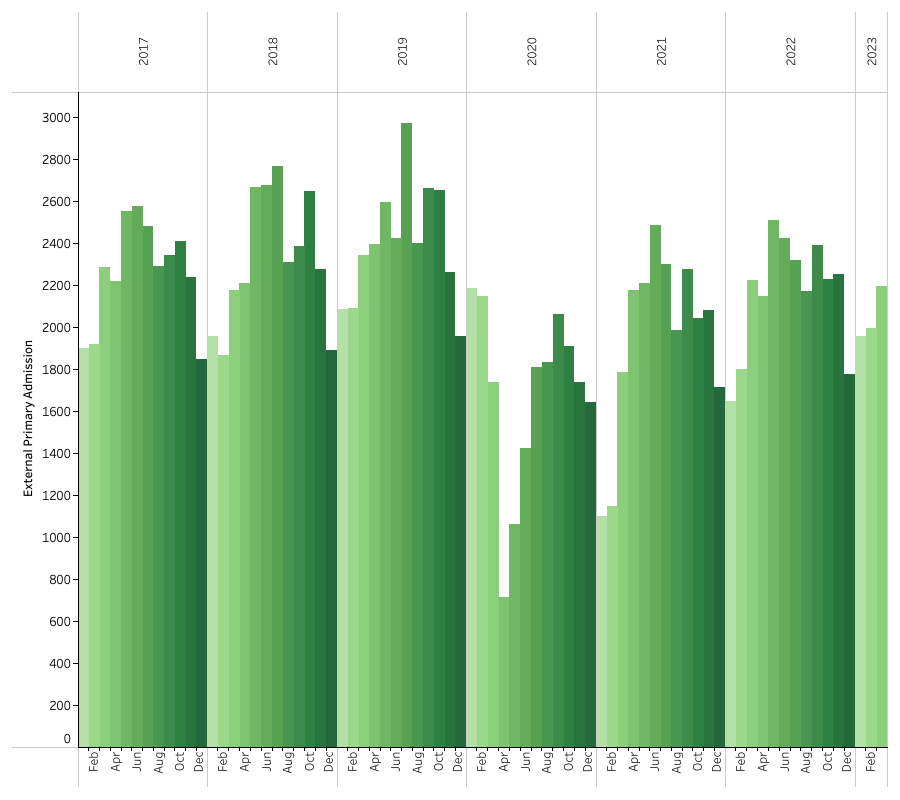


**Figure S3** Number of new diagnoses of T1DM within SUS by month in 0-17 year olds in England 2017 - 2023


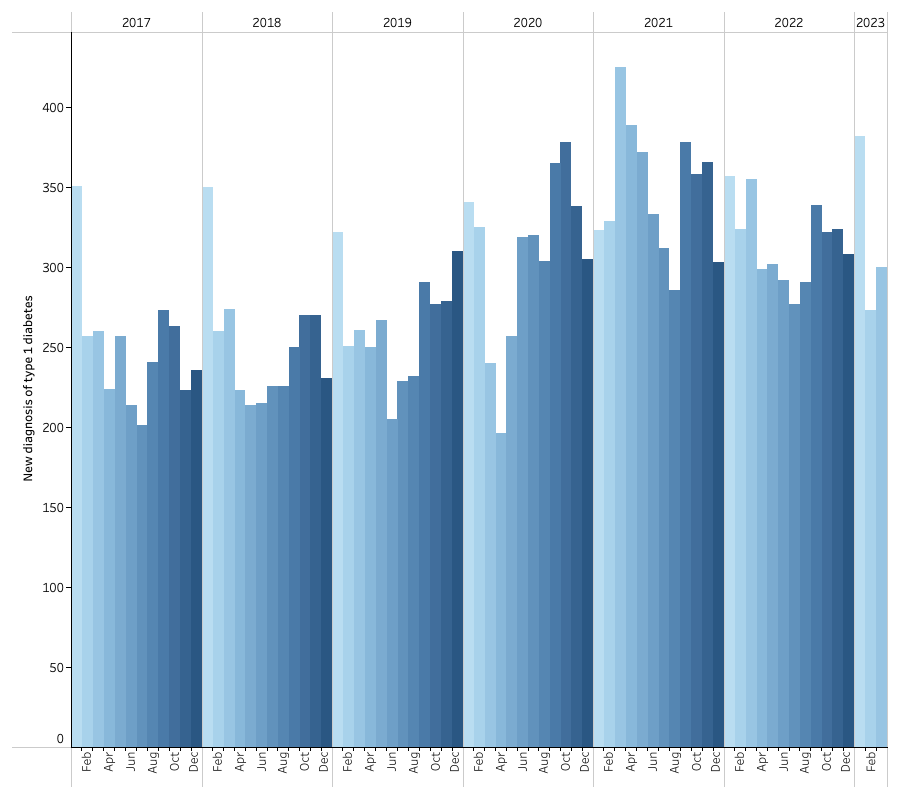


**Figure S4** Number of CYP with first positive test for SARS-CoV-2 within SUS by month in 0-17 year olds in England


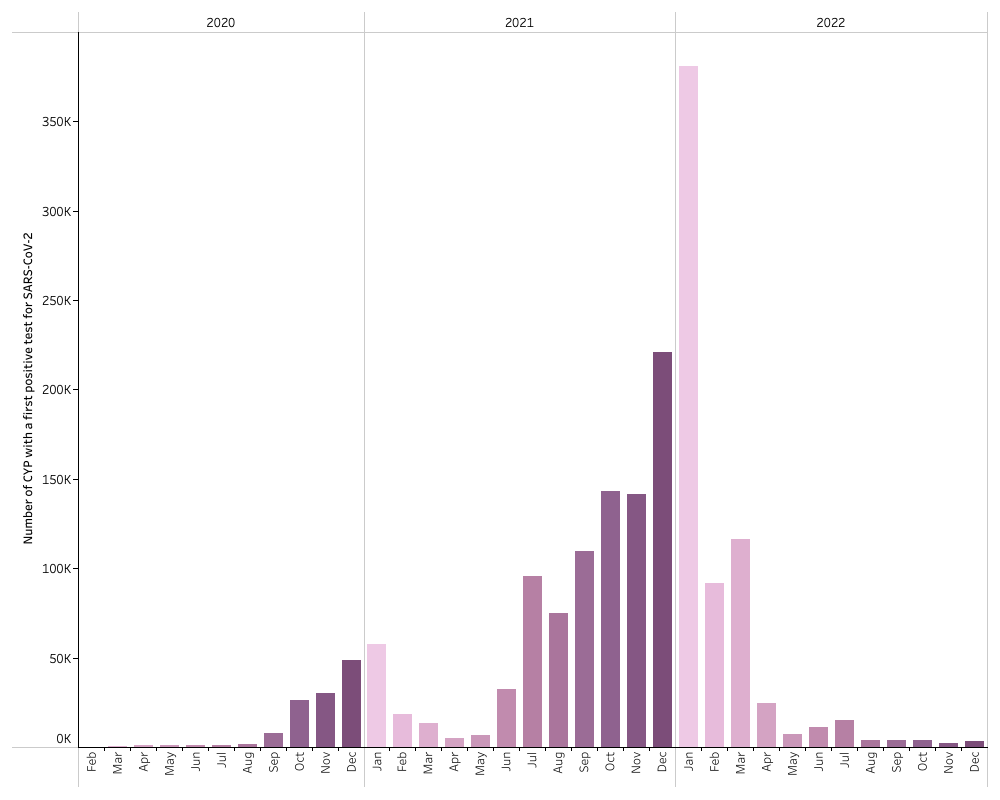


**Figure S5** Survival graphs showing probability of new diagnosis of type 1 diabetes in exposed compared with unexposed cohorts

**A.** New diagnosis probability in exposed cohort compared with trauma cohort

**
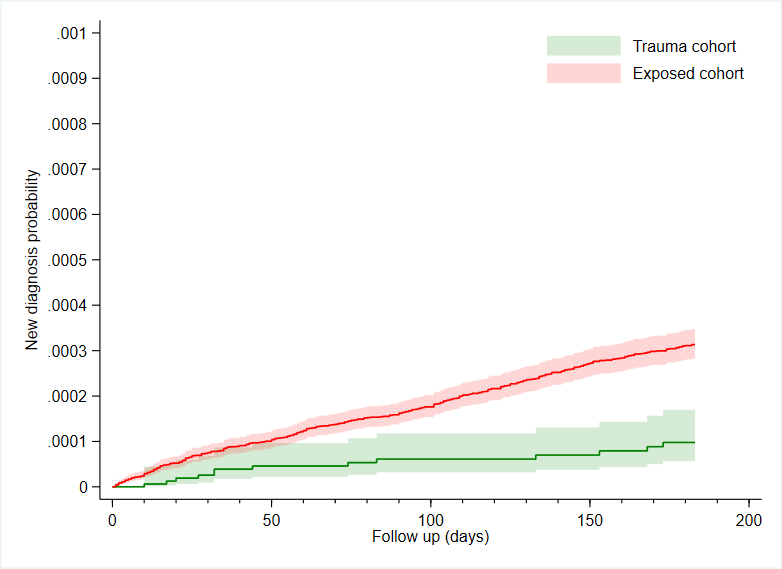
**

**B.** New diagnosis probability in exposed cohort compared with elective cohort


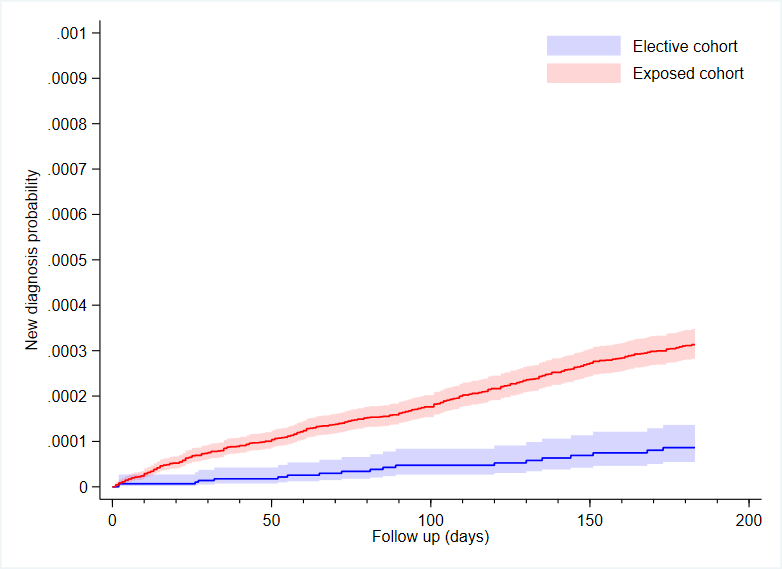


**C.** New diagnosis probability in exposed cohort compared with historic trauma cohort


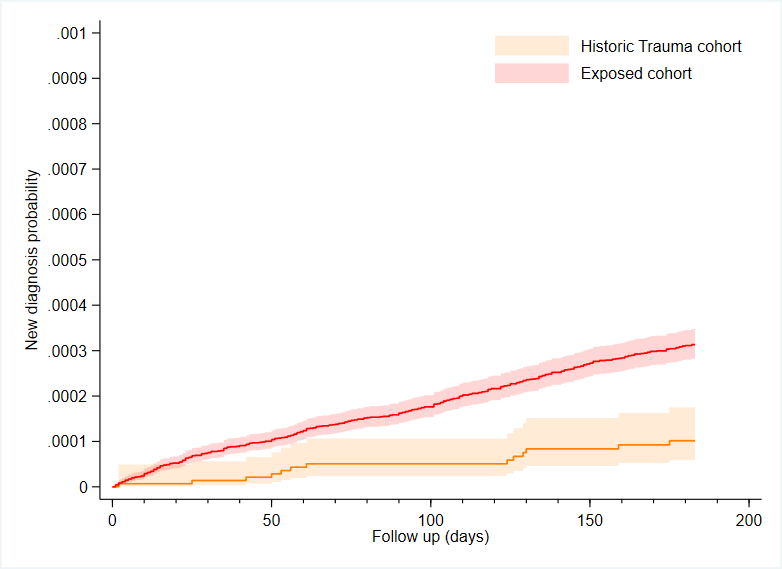


**D.** New diagnosis probability in exposed cohort compared with historic elective cohort


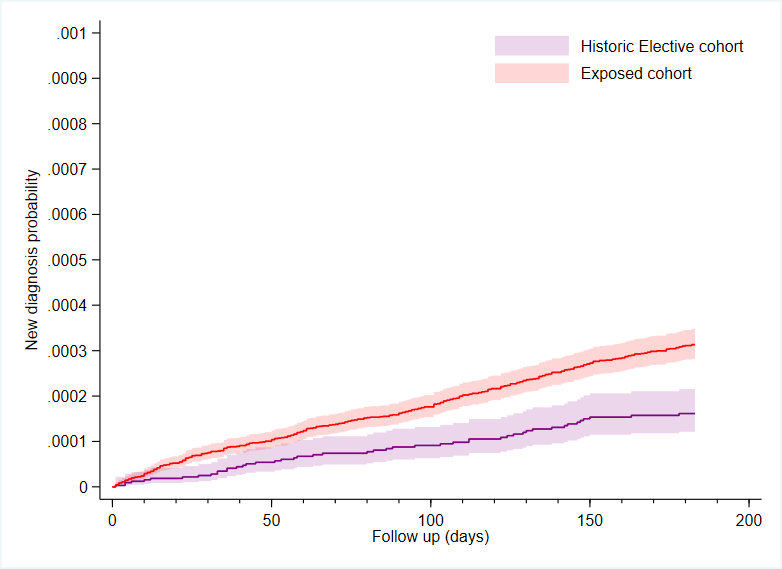


**E.** New diagnosis probability in exposed cohort compared with each unexposed cohort

**Figure S6** Incidence rate of new onset type 1 diabetes from day 28 to 208 days of follow up per 100,000 person-years of observation by dominant variant

**
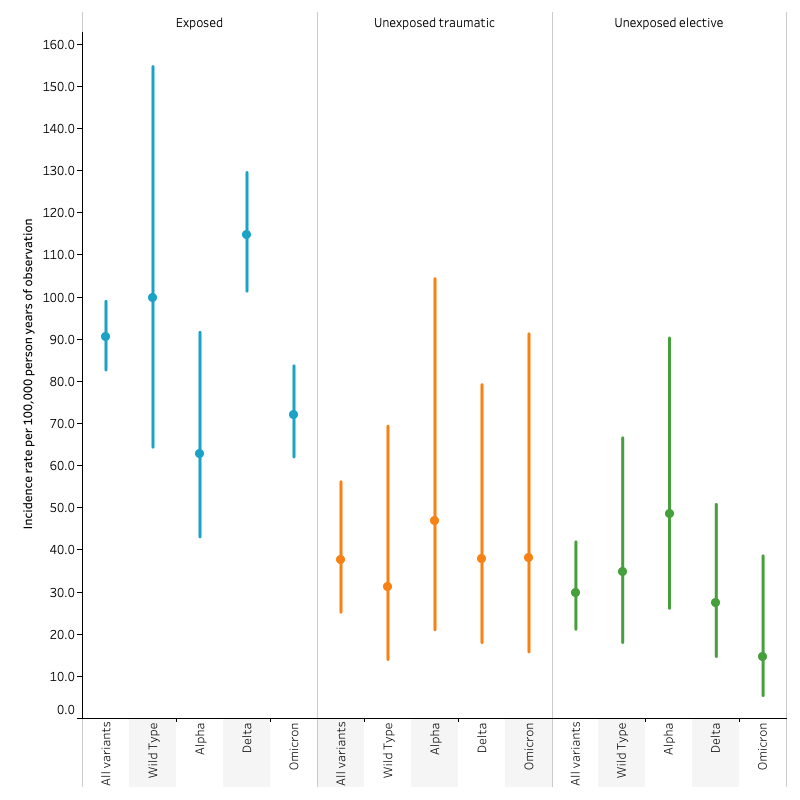
**
